# Environmentally Benign Formation of Nickel Hexacyanoferrate-Derived Mesoframes for Heterogeneous Catalysis

**DOI:** 10.3390/nano11102756

**Published:** 2021-10-18

**Authors:** Sascha Keßler, Elrike R. Reinalter, Johannes Schmidt, Helmut Cölfen

**Affiliations:** 1 Physical Chemistry, Department of Chemistry, University of Konstanz, Universitätsstrasse 10, D-78457 Konstanz, Germany; Sascha.Kessler@Uni-Konstanz.de (S.K.); Elrike.Reinalter@Uni-Konstanz.de (E.R.R.); 2 Department of Chemistry, Technical University of Berlin, Hardenbergstrasse 40, D-10623 Berlin, Germany; johannes.schmidt@tu-berlin.de

**Keywords:** nickel hexacyanoferrate, nanocrystals, mesocrystals, etching, heterogeneous catalysis, bisphenol-A, rhodamine-B

## Abstract

The tetramethylammonium hydroxide (TMAH)-controlled alkaline etching of nickel hexacyanoferrate (NiHCF) mesocrystals is explored. The alkaline etching enables the formation of hollow framework structures with an increased surface area, the exposure of active Ni and Fe sites and the retention of morphology. The ambient reaction conditions enable the establishment of a sustainable production. Our work reveals novel perspectives on the eco-friendly synthesis of hollow and colloidal superstructures for the efficient degradation of the organic contaminants rhodamine-B and bisphenol-A. In the case of peroxomonosulfate (PMS)-mediated bisphenol-A degradation, the rate constant of the etched mesoframes was 10,000 times higher indicating their significant catalytic activity.

## 1. Introduction

In today’s world, technology strives to develop innovative materials and methods to meet the daily needs of modern society. As overwhelming and fabulous as the ever-advancing path to perfection is, it also leaves its mark in the environment. In recent decades, there has been a significant increase in the pollution of freshwater systems. An ever-increasing number of contaminants such as carbamazepine, bisphenol-A and caffeine could be detected worldwide [1,2,3,4]. The effects of the increased occurrence of these contaminants can hardly be estimated today. However, since most of these contaminants are toxic to aquatic organisms, humans and wildlife, there is an urgent need for solutions to reduce them. Among others such as ozonation, adsorption or coagulation, one powerful technique to remove organic contaminants from aqueous systems is heterogeneous catalysis [5,6,7,8,9,10,11,12]. A widespread variant of this so-called wastewater treatment is PMS-activation on functional surfaces such as metal oxides or hydroxides (typically Ti, Mn, Fe, Co, Ni, Cu, Zn or Bi) [5,13,14,15,16]. The large surface area of nanomaterials contributes significantly to efficient PMS activation. Nevertheless, the production of such materials is often associated with high energy, the use of organic additives or organic solvents [6,13,14,15,17,18]. To meet the green chemistry aspects underlying wastewater treatment, environmentally friendly and energy-saving alternatives are crucial. An emerging class of materials that addresses these requirements are Prussian blue analogues (PBAs). These exhibit tremendous potential due to their structural diversity and benign manufacture. Typically, they are synthesized in water and at room temperature via a co-precipitation reaction of the precursors [19,20,21]. In general, this material class follows a simple structure principle which is represented by the following generic formula A_x_M[M′(CN)_6_]_y_⋅*n*H_2_O (0 ≤ x ≤ 2; 0 < y ≤ 1). Herein, M and M′ both are high spin and low spin transition metals (most commonly Mn, Fe, Co, Ni, Cu or Zn). A is considered as an associated alkali metal (e.g., Li, Na or K) to create neutrality of the compound. The cyano-ligands (CN^-^) bridge the transition metals M and M′. This structure allows the implementation of high functionality, as tailored pore sizes and a variety of different transition metals can be introduced. This means that PBAs are often used for applications such as water splitting, energy storage or wastewater treatment, or at least serve as starting material for them [22,23,24,25,26].

In the case of heterogeneous catalysis and the application of PBA-based nanomaterials as catalysts, they are often converted into metal oxides or hydroxides by thermal treatment, since the untreated PBA nanomaterials usually exhibit lower catalytic activities [22,26,27,28]. To avoid thermal treatment, but still obtain a material that exhibits high catalytic activity, increasing the active surface area is a useful method. Among other techniques, chemical etching represents an eco-friendly method for increasing the surface area as mostly ambient conditions are applicable. Most commonly, the etching can be mediated by acidic or alkaline agents [29,30].

In terms of heterogeneous catalysis, problems associated with the use of nanocrystals should not be ignored. As useful as the active surface may be for catalytic processes, the separation of the nanocrystals after catalysis can be problematic due to their small dimensions. Especially for large scale applications such as municipal wastewater treatment plants, it can be challenging to implement NC-based treatment approaches. Therefore, hierarchical structures are a sufficient means to combine the advantages of the properties of nanocrystals with easy separability. There are several approaches to implement NCs in hierarchical architectures. Often, they are immobilized on graphene or other carbon-derived materials such as sponges to form a nanocomposite [6,8,9,31,32,33]. On the other hand, superstructures can be formed by the spatial arrangement of NCs, which also promote their easy separability due to their size (i.e., micron-sized superstructures) [31,34]. In general, such systems containing NCs as building units can be characterized as colloidal crystals [35]. A special subspecies of these colloidal crystals are mesocrystals [36,37,38,39,40]. As soon as the NC subunits that build up a colloidal crystal are anisotropic and the alignment of those occurred on a long-range order, one can speak of a mesocrystal [41,42]. The nanoparticulate building blocks commonly can be aligned via bottom-up methods such as solvent evaporation or additive-mediated NC deposition (i.e., addition of a salt or an anti-solvent) [43,44,45,46]. However, the aforementioned processes mostly allow superstructures to be formed on substrates. Thus, it is difficult to use mesocrystals when their catalytic application requires homogeneous distribution in the medium. To circumvent this problem, colloidal superstructures can provide a resourceful alternative [47,48]. Especially, if the synthesis is carried out under sustainable and environmental aspects and the avoidance of complex and multi-stage routes. In literature, such systems have rarely been reported yet. Such in situ formations of colloidal superstructures are mostly achieved via micelle-mediated approaches [48].

Furthermore, the formation of colloidal hollow framework structures that are derived from mesocrystalline structures via chemical etching is hardly found in the literature. Retention of the mesocrystalline structural feature can be advantageous because the properties of mesocrystals exceed those of their bulk phase and are more similar to the properties of the corresponding nanocrystalline subunits [42]. If this is combined with the formation of an even larger surface area, physical properties such as mass transfer can be further enhanced and thus contribute to the improvement of catalytic properties [49].

In this work, we aimed at establishing the controlled alkaline etching of NiHCF mesocrystals to form highly ordered hollow framework structures with increased surface areas under ambient conditions. Owing to their mixed-composition of hexacyanoferrate and *β*-Ni(OH)_2_, high surface area and the facile separability, those structures were examined as catalyst for the PMS-activated degradation of the organic contaminants rhodamine-B and bisphenol-A. A rational investigation of the degradation parameters revealed that the NiHCF mesocrystal-derived hollow frameworks show high activity. Rhodamine-B can be degraded within 12 min which results in a rate constant of 0.41 min^−1^. In the case of bisphenol-A, the contaminant was removed after 8 min resulting in a rate constant of 0.26 min^−1^. The focus relied on NiHCF as starting material for the manufacture of the hollow frameworks due to its sustainable production, high water insolubility and promising functionalities. This work can potentially shed light on new perspectives to enable the transfer of this approach to other PBA systems and the implementation of various functional materials.

## 2. Materials and Methods

### 2.1. Chemicals

L-ascorbic acid (99.0%), nickel(II) acetate tetrahydrate (99.0%), trisodium citrate (≥99.0%), rhodamine-B (≥95.0%) and 4,4′-Isopropylidendiphenol (≥99.0%; bisphenol-A) were purchased from Sigma Aldrich (Taufkirchen, Germany). Potassium hexacyanoferrate(III) (98.0%) and tetramethylammonium hydroxide pentahydrate (98%) were purchased from Alfa Aesar (Kandel, Germany). Potassium peroxymonosulfate triple salt (PMS) and tert-butanol (99.0%) were purchased from Merck (Taufkirchen, Germany). Methanol (99.9%) was purchased from Carl Roth (Fontenay-sous-Bois, France)). Ethanol absolute (≥99.8%) was purchased from VWR (Fontenay-sous-Bois, France). All aqueous solutions and dispersions were prepared by using deionized Milli-Q water (18.2 mΩ cm^−1^) obtained from a Milli-Q Direct-8 system.

### 2.2. NiHCF Mesocrystal Synthesis

A mixture of nickel(II) acetate tetrahydrate (0.993 g, 3.993 mmol) and trisodium citrate dihydrate (2.114 g, 7.187 mmol) was dissolved in 40 mL of water. Potassium hexacyanoferrate(III) (0.873 g, 2.647 mmol) was dissolved in 40 mL of water. After both solutions were dissolved, the potassium hexacyanoferrate(III) solution was added using a peristaltic pump (20.0 mL min^−1^) to the nickel(II) acetate and trisodium citrate solution (40 mL). The mixture was stirred inside a double-walled reaction vessel equipped with a cryostat to crystallization temperature of 25 °C for 24 h. Afterwards, the resulted orange solid was purified by applying centrifugation at 7000 rpm for 10 min. Then, the supernatant was removed, and the precipitated mesocrystals were rinsed with water (4 × 30 mL). Subsequently, they were redispersed in water (sonication bath; 30 s) and the centrifugation was applied again (repetition: three times). The purified orange compound was dried in a vacuum oven at 40 °C for 24 h.

### 2.3. Alkaline Etching of the NiHCF Mesocrystals

In a typical procedure, 100 mg of NiHCF mesocrystals were dispersed in 60 mL of deionized water by using an ultrasonic bath. The dispersion was then transferred into a double-walled reaction vessel equipped with a cryostat to maintain a certain reaction temperature of 25 °C. Tetramethylammonium hydroxide pentahydrate (0.145 g, 0.800 mmol, 0.020 mol L^−1^) was dissolved in 40 mL of water. Subsequently, the aqueous tetramethylammonium hydroxide (TMAH) solution was added to the mesocrystal dispersion to initiate the alkaline etching process. The mixture was stirred for 24 h. The resulting green greenish solid was purified by centrifugation at 9000 rpm for 10 min. After removing the supernatant the precipitated mesoframes were rinsed with water (4 × 30 mL) and were re-dispersed in water (vortex mixer). Afterwards, the mesoframes were centrifuged again (repetition: three times). The purified green compound was dried in a vacuum oven at 40 °C for 24 h.

### 2.4. Rhodamine-B Degradation

10.0 mg of the respective catalyst (i.e., mesocrystals, Co_3_O_4_ or Mesoframes) was dispersed in 100 mL of an aqueous rhodamine-B solution (10 mg L^−1)^. To control the temperature (25 °C or 40 °C) of the degradation reaction, the as-prepared dispersion was transferred into a double-walled reaction vessel equipped with a cryostat. The dispersion was stirred for 30 min to maintain a certain reaction temperature. To initiate the degradation, 30.0 mg of PMS was added. To monitor the degradation progress, several aliquots were withdrawn at specific reaction times. After the catalyst was removed by applying a syringe filter (pore size: 0.25 µm), the supernatant was separated and transferred into a quartz cuvette (thickness: 1.0 mm) for UV-Vis analysis.

Catalyst and PMS impact on the degradation were examined by the variation of the respective concentrations. For the catalyst loading experiment, concentrations of 0.1, 0.2, 0.6 and 1.0 g L^−1^ were used. For exploring the PMS dosage, 0.1, 0.3, 0.7 and 1.5 g L^−1^ were used. The pH influence on the degradation reaction was studied by adjusting the pH to 3, 5, 7, 9 and 11. Therefore, hydrochloric acid or potassium hydroxide were used. Mechanistic investigations were conducted by using scavenger molecules. Here, aqueous bisphenol-A solutions contained 0.01 mol L^−1^ ascorbic acid (AA), 1.0 mol L^−1^ *tert*-butanol (*t*-BuOH) or 1.0 mol L^−1^ methanol (MeOH) during the respective degradation reaction.

### 2.5. Bisphenol-A Degradation

20.0 mg of respective particulate system (i.e., mesocrystals, Co_3_O_4_ or Mesoframes) were dispersed in 100 mL of an aqueous bisphenol-A solution (40 mg L^−1^). This dispersion was stirred in a double-walled reaction vessel equipped with a cryostat to maintain a certain starting temperature for the degradation (25 °C or 40 °C) for 30 min. When a constant temperature was reached, 20.0 mg of PMS was added to initiate the degradation reaction. Analysis of the concentration change of bisphenol-A during the degradation was maintained by taking aliquots at specific reaction times. For analysis of the sample, the catalyst had to be removed by applying a syringe filter (pore size: 0.25 µm). The filtered solution was transferred into a quartz cuvette (thickness: 1.0 mm) for UV-Vis analysis. For the impact of catalyst and PMS dosage on the bisphenol-A degradation, the respective concentrations were varied. For the catalyst loading experiment, concentrations of 0.2, 0.4, 1.2 and 2.0 g L^−1^ were used. In the case of PMS, 0.2, 0.4, 1.0 and 2.0 g L^−1^ were used. Regarding the pH influence on the degradation performance, the pH was adjusted to 3, 5, 7, 9 and 11 by using hydrochloric acid or potassium hydroxide. In order to investigate the mechanism, scavenger experiments were conducted by using aqueous bisphenol-A solutions containing 1.0 mol L^−1^ *t*-BuOH or 1.0 mol L^−1^ ethanol (EtOH) during the degradation reaction.

### 2.6. Analytics

Brunauer–Emmett–Teller surface area measurements: Surface area was determined by measuring nitrogen sorption isotherms at 77 K by an Autosorb-iQ-MP from Quantachrome (Boynton Beach, FL, USA) and a Quadrasorb SI (Boynton Beach, FL, USA) device. The obtained data was evaluated by means of the BET theory. For the BET surface area measurements, the mesocrystals or frameworks were dried at 60 °C under vacuum overnight. Then, the NCs were transferred into the measurement cell. Electron microscopy: Scanning electron microscopy micrographs were recorded by using a Gemini 500 (Oberkochen, Germany) provided by Zeiss operating at 5 kV. The Gemini 500 is able to detect secondary and backscattered electrons. High quality micrographs can be obtained due to a drift-compensated frame-averaging mode (avoiding charging effects). Energy-dispersive X-ray spectroscopy was also applied by the Gemini 500 operating at 15 kV. In addition, EDS spectra were also recorded on a Hitachi TM3000 Tabletop SEM (Chiyoda, Japan) using 15 kV acceleration voltage and a Quantax EDX detector. The samples were prepared by drop-casting a dispersion on a double-polished Si (100)-wafer. The dried sample was then coated with a gold film (thickness: approximately 4.0 nm). A Zeiss Libra 120 EF-TEM (Oberkochen, Germany) operating at an acceleration voltage of 120 kV provided the transmission electron microscopy (TEM) micrographs. Fourier-transform Infrared spectroscopy: Transmission infrared spectroscopy was conducted by using a Perkin Elmer device (Spectrum 100 FTIR, Schwerzenbach, Switzerland). For using the transmission mode, potassium bromide pellets have to be produced by mixing 0.5 g of potassium bromide with the product compound. After the milling of this mixture, the powder is pressed into a pellet with 5 t for 30 min. Powder X-ray diffraction characterization was performed using a Bruker D8 (Bellerica, MA, USA) Advance equipped with a scintillation counter, and a Bruker D8 (Bellerica, MA, USA) Discovery with a Lynxeye XE detector. UV-Vis spectroscopy was performed with a Varian Cary 50 (Palo Alto, CA, USA) spectrometer equipped with a 1.5 nm fixed spectral bandwidth and full spectrum Xe pulse lamp single source. A quartz cuvette with a thickness of 1.0 mm was used in every experiment. X-Ray photoelectron spectra were conducted on a K-Alpha™ + X-ray Photoelectron Spectrometer System provided by Thermo Scientific (Waltham, MA, USA). It is equipped with a Hemispheric 180° dual-focus analyzer with 128-channel detector and used an X-ray monochromator with micro focused Al-K_α_ radiation.

## 3. Results and Discussion

Herein, we describe the formation of NiHCF mesocrystals via the previously published additive-mediated co-precipitation method (Equation (1)) [21].

3Ni^2+^ + 2K_3_Fe(CN)_6_ ⇄ 6K^+^ + Ni_3_[Fe(CN)_6_]_2_ ↓ (1)

**Figure 1:**
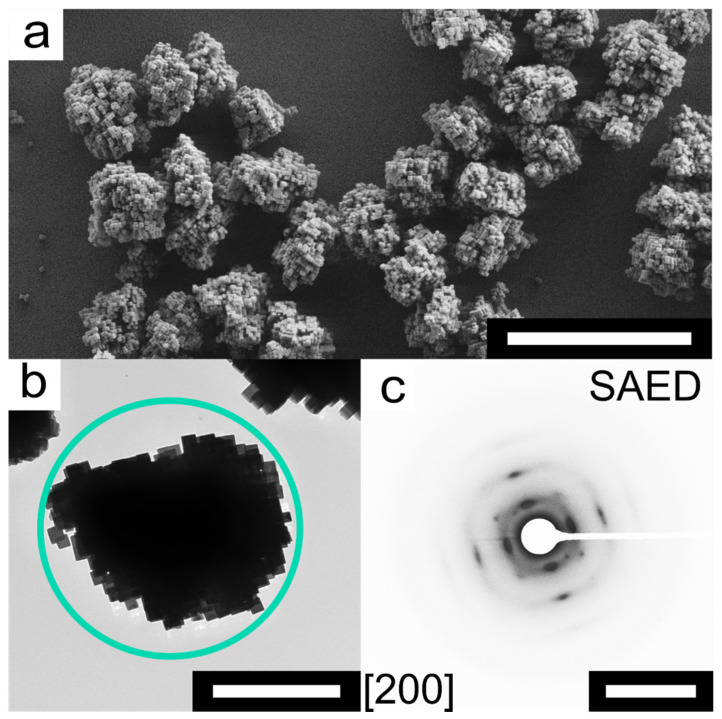
Analysis of NiHCF mesocrystal morphology. (**a**) SEM micrograph of NiHCF mesocrystals. (**b**) TEM micrograph of an individual NiHCF mesocrystal. (**c**) SAED pattern that corresponds to the individual mesocrystal (Diffraction area indicated by the green square; view from [200] zone axis). Scale bars: 5.0 µm (top), 1.0 µm (bottom left) and 5.0 nm^−1^ (bottom right).

The mesocrystals can be synthesized by direct mixing of the precursor solutions within a short time period of about 24 h. Equal volumes of two aqueous solutions containing potassium hexacyanoferrate(III) (0.02 mol L^−1^) and nickel(II) acetate (0.03 mol L^−1^) were mixed for this purpose. As crystallization regulating agent, trisodium citrate has so far proven to be most suitable. The morphology of the synthesized NiHCF mesocrystals was characterized via scanning electron microscopy (SEM). The SEM analysis shows that the obtained superstructures are composed of individual cubic shaped nanocrystals (NCs). Furthermore, almost all NCs are incorporated into the superstructures and even after ultrasound-treatment these superstructures retain their habitus (Figure 1a). Transmission electron microscopy (TEM) analysis shows the ordered structure of the mesocrystals at a higher magnification (Figure 1b). Selected area electron diffraction (SAED) of an individual mesocrystal shows the presence of sharp reflexes only with slight arcs indicating the high order within the superstructure (Figure 1c). Typically, the term mesocrystal describes an ordered superstructure composed of anisotropically shaped NCs [41]. The mutual alignment of the NCs within the superstructure is confirmed by the occurrence of sharp Bragg peaks implying a long-range order on the atomic scale (i.e., the peaks can be compared to those of a single crystal) [42,45]. Powder X-ray diffraction (PXRD) analysis confirmed the crystallinity of the obtained mesocrystals and the preferred *F*$\bar{4}$3*m* space group (Figure S1). Energy-dispersive X-ray spectroscopy (EDS) revealed a ratio of 1.24 between Ni^II^ and Fe^III^ ions which is characteristic for NiHCF systems with an ideal formula of Ni_3_[Fe(CN)_6_]_2_ (Figure S1, Table S1). In addition, the Brunauer–Emmett–Teller (BET) surface area was determined to be 35 m^2^ g^−1^ (Figure S2).

**Figure 2:**
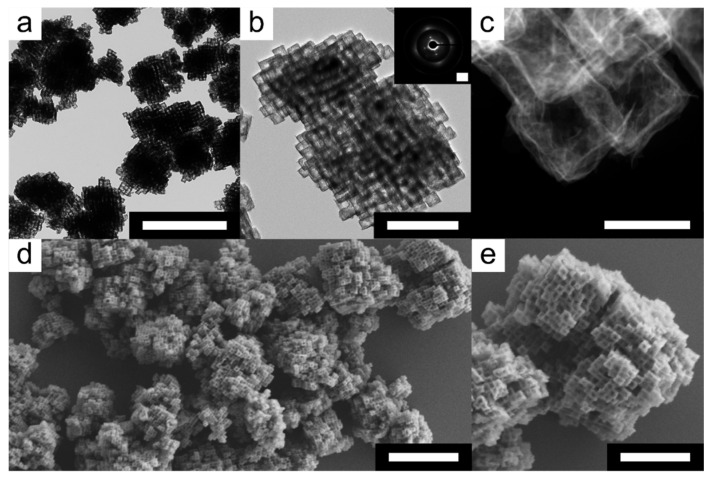
Morphological characterization of the hollow frameworks after alkaline etching. (**a**) Low and (**b**) high magnification TEM micrographs of NiHCF mesocrystal-derived hollow frameworks and the corresponding SAED pattern (inset; from the [200] zone axis) obtained from TMAH-mediated etching at room temperature in aqueous media. (**c**) Dark-field HRTEM micrograph of the NCs on the surface of a hollow framework. (**d**) SEM micrograph of hollow frameworks distributed on a silicon (100) wafer. (**e**) High magnification SEM micrograph of a hollow framework. Scale bars: 2.0 µm (top left), 0.5 µm (top middle), 5.0 nm^−1^ (inset); 100 nm (top right), 2.0 µm (bottom left) and 1.0 µm (bottom right).

Previous studies demonstrated that the NiHCF mesocrystals proved to be an easily accessible and effective catalyst for the removal of caffeine [50]. In particular, the easy removal of the catalyst by filtration due to its size of 2–3 µm represents a significant advantage over NCs. In general, an essential feature to improve the catalytic activity of heterogeneous catalysts is the increase in the surface area. Especially, the formation of many active sites is crucial to achieve an increase in activity. The easiest way to obtain a higher surface is to hollow out structures via etching. In our case, the alkaline etching of the NiHCF mesocrystals by using tetramethylammonium hydroxide (TMAH) appeared to be a facile and powerful process to obtain hollow frameworks. Therefore, the NiHCF mesocrystals were dispersed in deionized water at room temperature and an aqueous solution of TMAH (0.02 mol L^−1^) was subsequently added. After 23 h, the etched superstructures were purified via centrifugation and washing. The morphology of the etched superstructures was analyzed by TEM. Low magnification analysis shows the formation of hollow superstructures that retained their original habitus (Figure 2a). Almost no individual NCs that could have been broken out of the superstructures by etching were observed. It appears that the alkaline etching did not affect the mutual alignment of the NCs within the superstructures. TEM analysis at high magnification shows that the hollow frameworks obtained still consist of individual NCs, which also confirms that the NiHCF mesocrystals also consist of individual NCs inside (Figure 2b). SAED of an isolated hollow framework confirmed that the individual NCs kept their common crystallographic orientation due to the occurrence of sharp reflexes with slight arcs (Figure 2b, inset; from the [200] zone axis). This means the characteristics of a mesocrystal type I can be transferred to the hollow framework [41]. These results demonstrate the successful formation of mesocrystalline frameworks (mesoframes) obtained from alkaline etching of NiHCF mesocrystals. High-resolution dark-field TEM (HRTEM) analysis reveals that the individual NCs on the surface of the mesoframes are almost hollow and only an insoluble cubic shell remained after etching (Figure 2c).

The TEM investigations could show a hollow architecture of the NCs inside the superstructure, in addition the SEM analysis could reveal that the NCs exhibit some voids and cavities on their surfaces (Figure 2d). In particular, SEM analysis of the NCs surfaces at higher magnification could show a more detailed view of the voids and cavities created by etching. (Figure 2e). The BET surface area for the hollow frameworks is 96 m^2^ g^−1^, 2.7 times higher than that of the NiHCF mesocrystals (Figure S3).

**Figure 3:**
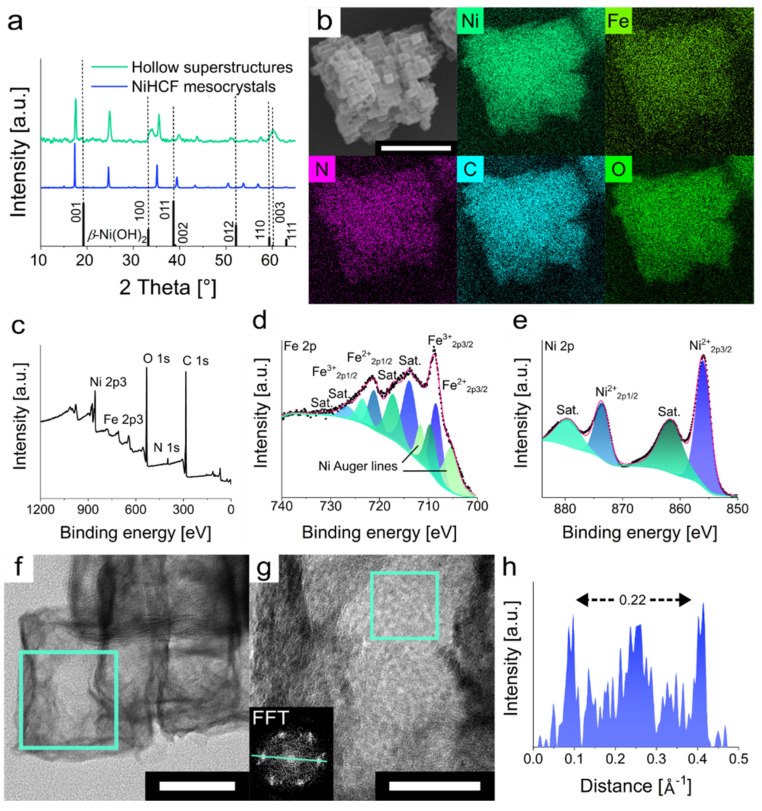
Analysis of material composition after alkaline etching. (**a**) Normalized PXRD pattern of NiHCF mesocrystals and mesoframes (COD reference number for *β*-Ni(OH)_2_: 1011134).50 (**b**) EDS mapping of the elements (Ni, Fe, N, C, and O) within the NiHCF-derived hollow superstructures. (**c**) XPS total survey of the NiHCF-derived hollow superstructures. XPS spectrum of the mesoframes for (**d**) Ni 2p and (**e**) Fe 2p. (**f**) HRTEM microscopy at low magnification from one NC (inset shows the area for higher magnification analysis). (**g**) HRTEM microscopy with high magnification and the corresponding FFT of the green square area. (**h**) Line profile of the FFT for analysis of the mesoframe lattice structure (indicated by the green line within the FFT). Scalebars: 50 nm (bottom right) and 20 nm (bottom middle).

To verify the composition of the material, PXRD analysis of the mesoframes was conducted. Besides the appearance of the original NiHCF mesocrystal reflexes, one new intense reflex at 33.8° and four smaller reflexes at 18.8, 38.4, 51.6 and 60.6° emerged (Figure 3a). The four small reflexes can only be observed at a higher magnification, as they partially overlap with the NiHCF reflexes (Figure S4a). These newly emerged reflexes can be assigned to *β*-Ni(OH)_2_ as they show similar reflex positions (Figure S4b) [51]. The original NiHCF reflexes appear due to remaining NiHCF within the hollow superstructures. Analysis of the element distribution via EDS mapping confirmed the homogeneous distribution of Ni, Fe, N, C and O within the hollow superstructure (Figure 3b). Especially, the presence of oxygen and the lack of intense signals for Fe and N confirms a HCF-replacement by hydroxide ions (i.e., EDS mapping and point analysis for NiHCF mesocrystals shows a higher intensity for Fe and N, and almost no signal for O; Figures S1, S5 and S6). The [Ni]:[Fe] ratio also increased from 1.43 of the NiHCF mesocrystals to 4.06 for the hollow superstructures (Figure S6 and Table S2). In addition, FT-IR spectroscopy was applied to further explore the materials composition after the alkaline etching (Figure S7). The FT-IR spectrum of original NiHCF mesocrystals shows the expected signals at 3650, 3400 and 1615 cm^−1^ that can be assigned to hydroxyl groups on the mesocrystal surface and interstitial water molecules. The two intense signals at 2166 and 2100 cm^−1^ are attributed to the stretching vibration band of the cyano group (C≡N) and its bridging isomer coordinated with Ni^II^ and Fe^III^ [52]. The FT-IR spectrum of the etched hollow superstructures, on the other hand, reveals the disappearance of the sharp signal at 3650 cm^−1^. The signals for the cyano group are redshifted to 2097 and 2055 cm^−1^ and an additional signal at 2011 cm^−1^ emerged which may correspond to a stretching vibration of a cyano group coordinated to Fe^II^ [30]. The signals at 1478, 1370 and 650 cm^−1^ can be assigned to *β*-Ni(OH)_2_ [53]. To further validate the hypothesis of the formation of *β*-Ni(OH)_2_, X-ray photoelectron spectroscopy (XPS) was conducted. The survey spectrum of the hollow superstructures shows coexistence of Ni, Fe, N, C and O (Figure 3c). In particular, the intense signal for oxygen and the almost disappeared signal for nitrogen compared to the signals of the measurement spectrum for the NiHCF mesocrystals support the results of the EDS analysis (Figure S11). The high-resolution Fe 2p spectrum shows binding energies (BEs) at 708.6 and 721.2 eV that can be assigned to Fe^II^ 2p_3/2_ and Fe^II^ 2p_1/2_, respectively (Figure 3d). The signals at 714.1 and 726.7 eV can be identified as shake-up satellites (sat.), which are characteristic for Fe^II^. The BEs at 709.8 and 723.6 eV can be ascribed to Fe^III^ 2p_3/2_ and Fe^III^ 2p_1/2_ and the signals at 717.4 and 731.1 eV are the corresponding shake-up satellites. The two signals with BEs of 705.6 and 712.0 can be ascribed as Auger-lines of the present nickel [54]. In principle, the BEs found correspond to typical NiHCF BEs (Figure S8) [55]. In the case of the high-resolution Ni 2p spectrum, the two signals with BEs of 856.0 and 873.8 eV that can be assigned to Ni 2p_3/2_ and Ni 2p_1/2_ of *β*-Ni(OH)_2_ (Figure 3e) [56]. The two signals at 861.9 and 879.7 eV can be ascribed as shake-up satellites. In addition, high-resolution transmission electron microscopy (HRTEM) analysis showed the presence of lattice fringes with an interplanar spacing of 0.228 nm derived from the fast Fourier transformation (FFT) (Figure 3f,g). This interplanar spacing may correspond to the [002] plane of *β*-Ni(OH)_2_ [50]. These results together with the XPS, FTIR, EDS and PXRD characterizations point to the reasonable synthesis of a hexacyanoferrate-*β*-Ni(OH)_2_ mixed compound.

**Figure 4:**
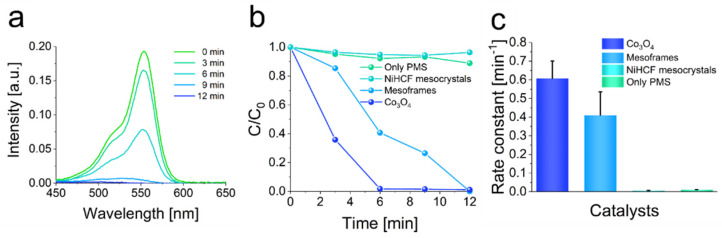
Degradation of rhodamine-B by using mesoframes for PMS-activation. (**a**) UV-Vis absorption spectra of rhodamine-B degradation within 12 min. (**b**) Degradation efficiencies of using Co_3_O_4_, mesoframes and NiHCF mesocrystals as catalysts. The efficiency in the absence of any catalyst is also illustrated. (**c**) Rate constants derived from the respective degradation curves. Reaction conditions: [Rhodamine-B] = 10.0 mg L^−1^, V = 100 mL, T = 25 °C, pH = 5, [Mesoframe] = 0.10 g L^−1^, [PMS] = 0.30 g L^−1^.

The TMAH-mediated etching of NiHCF mesocrystals resulted in the formation of colloidal mesoframes. These were easily dispersed in aqueous media resulting in a homogeneous distribution. In addition, the increase in material surface area can be beneficial for enhancing catalytic performance in heterogeneous catalysis of organic pollutant degradations. To explore the effect of activity enhancement, the PMS-activated degradation of rhodamine-B using different catalysts such as NiHCF mesocrystals, mesoframes and Co_3_O_4_ was conducted (Figure 3b and Figure S9). To monitor the degradation reaction in the presence and absence of the different catalysts, UV-Vis spectroscopy was applied. The degradation of rhodamine-B in the absence of any catalyst and in the presence of NiHCF mesocrystals shows that the PMS-activation is delayed compared to degradation in the presence of mesoframes or Co_3_O_4_ (Figure 4a). The enhancement of the catalytic activity may be explained by the formation of hydroxy and sulfate radicals on the larger surface of the mesoframes. Even more, the etching process may lead to the formation of voids and vacancies which expose more Ni^II^ and Fe^II/III^-containing active sites. From the mechanistic point of view, the presence of suitable scavengers for the possibly formed radicals can hinder or at least delay the degradation of the contaminant. In this case, ascorbic acid (AA) was chosen as scavenger for hydroxy radicals and sulfate radicals. In addition, *tert*-butanol (*t*-BuOH) was chosen for only hydroxy radicals as well as methanol (MeOH) only for sulfate radicals. By applying the scavengers, their contributions were derived from their rate constants (Figures S10 and S11). When MeOH was used to quench the degradation, only 31% of rhodamine-B was degraded after 12 min. In terms of t-BuOH, 75% was degraded after 12 min (Figures S10 and S11, Table S3). It appears that the degradation of rhodamine-B is mainly driven by the presence of hydroxyl radicals as the degradation in the presence of MeOH was more delayed compared to the degradation in the presence of t-BuOH.

By applying the NiHCF-derived mesoframes as a catalyst, approximately 99% of the rhodamine-B was removed after 12 min, resulting in a rate constant of 0.41 min^−1^ (Figure 4b,c, Table S3). The degradation in the presence of Co_3_O_4_-powder shows a slightly better performance, as 98% of the rhodamine-B was removed after 6 min leading to a rate constant of 0.61 min^−1^ (Figure 4b,c). The catalytic activity of the respective catalysis is characterized by its reaction kinetics and the derived rate constant. In the case of the rhodamine-B removal, the pseudo first-order kinetic model using the first-order rate equation (Equation (2)) was applied to derive the rate constant.

C_t_ = C_0_ ∙ exp(−k/t) (2)

herein, k represents the first-order reaction rate, t can be ascribed as the degradation time, C_0_ is the initial and C_t_ the time-dependent rhodamine-B concentration. These results suggest that the mesoframes are a reliable candidate for the oxidative degradation of rhodamine-B. Due to its high rate constant, this catalyst can be ranked among the most common catalysts for heterogeneous catalysts such as Co_3_O_4_. Besides the general removal of the contaminant, the process parameters affecting the degradation efficiency such as catalyst-loading, PMS dosage, effect of pH and temperature influence were examined (Figures S12–S17, Table S3). In the case of the PMS dosage, it was found that a moderate dosage was already used (Figure S14). The pH influence showed a distinct increase in activity with increasing the pH value (Figures S15 and S16). The reaction rate was increased and the activation energy was determined by raising the reaction temperature. At an elevated temperature of 40 °C, a 6.4 times higher rate constant of 2.63 min^−1^ was obtained (Figures S12d and S17 and Table S3). By applying the Arrhenius equation (Equation (3)), the activation energy E_A_ was calculated to 96.3 kJ mol^−1^ considering the rate constants obtained at 25 and 40 °C.

k = A ∙ exp(−E_A_/RT) (3)

wherein, k is the rate constant, A can be described as the pre-exponential, R is the universal gas constant and T is the absolute temperature.

**Figure 5:**
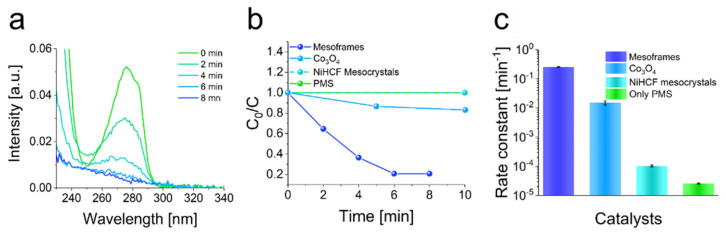
PMS-mediated degradation of bisphenol-A using mesoframes as catalyst. (**a**) UV-Vis absorption spectra of time-dependent bisphenol-A degradation. (**b**) Degradation efficiencies of bisphenol-A in the presence of the mesoframes, Co_3_O_4_, NiHCF mesocrystals and in the absence of any catalyst. (**c**) Rate constants that correspond to the respective bisphenol-A removals (logarithmic scale for the illustration of all rate constants). Reaction conditions: [Bisphenol-A] = 40.0 mg L^−1^, V = 100 mL, T = 25 °C, pH = 7, [Mesoframe] = 0.20 g L^−1^, [PMS] = 0.20 g L^−1^.

In principle, the efficient removal of rhodamine-B by the mesoframes showed that they appear to be promising as heterogeneous catalysts. In particular, the cost-effective and low-energy production of the frameworks and the very simple separation of the catalyst by filtration are notable. To emphasize the practical use of those superstructures, the mesoframes were used to degrade the plasticizer and contaminant bisphenol-A. Therefore, the mesoframes were dispersed in an aqueous solution containing bisphenol-A (40 mg L^−1^). The degradation was initiated by the addition of PMS. The degradation reaction was monitored by UV-vis spectroscopy, as in the case of the rhodamine-B degradation reaction. The mesoframes showed a very high activity in the degradation reaction. Most of the bisphenol-A was degraded after only 8 min, resulting in a rate constant of 0.26 min^−1^ (Figure 5a). This is more than 10,000 times higher than the rate constant of PMS and thus a very significant increase in catalytic activity. In comparison, a rate constant of only 0.02 min^−1^ was obtained for the bisphenol-A removal in the presence of Co_3_O_4_. The degradation in the presence of NiHCF mesocrystals or in the absence of a catalyst, on the other hand, hardly differed, in that almost no PMS activation occurred and catalysis was thus initiated (Figure 5b,c and Figure S18 and Table S4). The improvement of the catalytic activity compared to the NiHCF mesocrystals may also correspond to the availability of a higher surface area and the increased occurrence of small cavities and the associated exposure of active Fe^III^ and Ni^II^ ions, as described in the analysis of the composition. To create a link to the mechanistic details, scavenger molecules were also used to examine the impact of certain radical species. In this case, ethanol (EtOH) was used as suitable scavenger for sulfate and hydroxy radicals and *t*-BuOH was chosen to be suitable for hydroxy radicals. The respective contributions and their rate constants were derived from the corresponding kinetic data of the degradation experiments in the presence of the respective scavenger (Figures S19 and S20, Table S4). For the use of EtOH as scavenger, 70% of the bisphenol-A could be degraded after 6 min whereas 79% was degraded when *t*-BuOH was used. Based on these data, the degradation seems to be caused preferentially by hydroxyl radicals, but sulfate radicals also have a significant share in it. For a practically relevant system such as that of bisphenol-A, it is also necessary to investigate the kinetic parameter-determining factors in order to gain a better understanding of the catalytic processes. To verify the data obtained from the rhodamine-B removal, as well as the impact of catalyst-loading, PMS dosage, pH and temperature on the degradation reaction was investigated (Figures S20–S25). By raising the catalyst and PMS dosage, the reaction rate drastically increased (Figures S21 and S22). As for the influence of pH, higher pH values favored the degradation reaction, while lower pH values decreased the reaction rate similar to rhodamine B degradation (Figures S23 and S24). The activation energy EA was also calculated via the Arrhenius equation (Equation (3)) to a value of 44.1 kJ mol^−1^ (Figures S20d and S25). For this, the rate constants obtained by means of degradation reactions at 25 °C and 40 °C were used (Table S4).

These results indicate that the catalytic activity of the mesoframes is comparable or even higher than that of the most recently published catalysts or the commercial Co_3_O_4_ catalyst (Table S5) [8,16,18,33,57,58,59]. A major advantage over all these catalysts is that the mesoframes are produced at only 25 °C, whereas the other catalysts usually require high temperatures for production (i.e., temperatures between 80 °C and 650 °C were applied).

**Figure 6:**
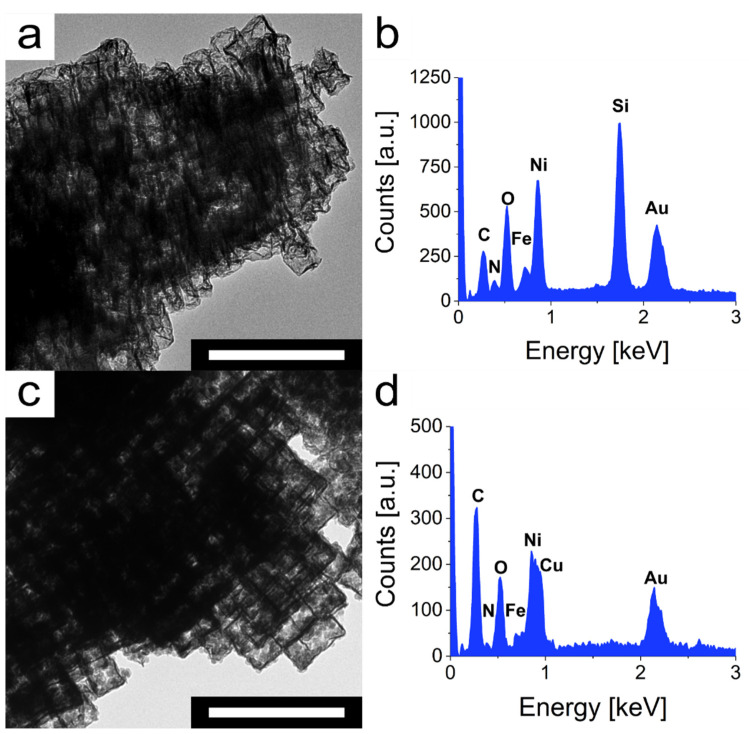
Analysis of the mesoframes morphology and composition after catalysis. (**a**) TEM micrograph of the mesoframes after rhodamine-B removal. (**b**) EDS spectrum of mesoframes after the catalysis. Reaction conditions: [Rhodamine-B] = 10.0 mg L^−1^, V = 100 mL, T = 25 °C, pH = 5, [Mesoframe] = 0.10 g L^−1^, [PMS] = 0.30 g L^−1^. (**c**) TEM micrographs of the mesoframes after bisphenol-A catalysis. (**d**) Corresponding EDS spectrum of mesoframes after the bisphenol-A catalysis (Copper signal obtained from analyzed TEM mesh). Reaction conditions: [Bisphenol-A] = 40.0 mg L^−1^, V = 100 mL, T = 25 °C, pH = 7, [Mesoframe] = 0.20 g L^−1^, [PMS] = 0.20 g L^−1^. Scale bars: 500 nm.

In general, a catalyst should emerge unchanged after use. To validate whether this applies to the mesoframes, their morphology and composition were examined for changes after the respective decomposition reactions of rhodamine-B and bisphenol-A. TEM analysis of the catalysts after the heterogeneous catalysis showed, especially for rhodamine-B, that the mesoframes are damaged in places and surrounded by fragments (Figure 6a). In the case of bisphenol-A, this is not observed with such intensity (Figure 6c). Nevertheless, in both cases, the framework structure is still intact, which shows that the catalysis process had only a minor impact on the morphology. For the influence of catalysis on the composition and especially the associated ratio of Ni^II^ to Fe^III^ ions, only a minor influence was detected by EDS analysis of the catalysts after catalysis. After the catalysis of rhodamine-B, the ratio between Ni^II^ to Fe^III^ changed from 4.06 to 4.41 (Figure 6b, Table S6). For the catalysis of bisphenol-A, a change from 4.06 to 3.98 was detected (Figure 6d, Table S7). It can be seen that for both samples a certain decrease or increase in Ni^II^ to Fe^III^ can be observed, which is within the range of measurement accuracy. The differences in the ratios can occur due to leaching or due to different etching progress during the etching process of the mesocrystals. Owing to their partly different sizes, smaller structures can be etched more strongly than larger ones. In addition, the TEM micrographs demonstrate that most of the structures are intact, so that a major occurrence of leaching can be ruled out. Taking these results into account, the mesoframes can certainly be considered as efficient heterogeneous catalysts for the degradation of rhodamine-B and especially the contaminant bisphenol-A.

## 4. Conclusions

In summary, we have investigated the TMAH-induced alkaline etching of NiHCF mesocrystals to fabricate mesocrystal-derived mesoframes. It was found that the TMAH etches small cavities and voids into the architecture of the mesocrystals, while the mesocrystalline character is preserved as the architecture still consists of individual NCs and shows slight arcs in the SAED pattern. This approach then leads to a 2.7-fold increase in surface area to 96 m^2^ g^−1^. Nevertheless, the mesoframes still exhibit a rigid architecture. The analysis of the composition showed that after the etching process, a mixed phase between hexacyanoferrate and *β*-Ni(OH)_2_ was formed. A key feature that corresponds to this approach, and to which attention should be paid, is that the mesoframes are produced in a low-energy and environmentally friendly way, as the etching process is carried out at room temperature and in water. Even more, due to the simplicity, reliability, and reproducibility of the process, it was possible to establish a sustainable manufacture of mesoframes. By taking into account their dimensions of 2–3 µm, the mesoframes can be very easily filtered out of dispersion, making them ideal for heterogeneous catalysis of contaminants. We demonstrated the successful and efficient removal of rhodamine-B and bisphenol-A by applying the NiHCF mesocrystal-derived mesoframes. They can remove 99% of rhodamine-B within 12 min resulting in a rate constant of 0.41 min^−1^. In the case of bisphenol-A, after 8 min almost the entire contaminant was degraded which classifies the catalyst as very powerful, considering its benign manufacture.

The comparison with Co_3_O_4_ also showed that the performance of the mesoframes can certainly keep up with that of a conventional catalyst. For the mesoframes, a rate constant of 0.26 min^−1^ was determined, which is 1.7 times higher than that determined for Co_3_O_4_. The enhancement of the activity seem to emerge from the exposure of the Fe^II^ and Fe^III^ active sites alongside the presence of *β*-Ni(OH)_2_. The catalyst shows, for both systems, reasonable stability, as no major issues regarding ion leaching or dissolution could be observed. Thus, we believe that this work could shed light on new perspectives for practical applications of PBA-based functional catalysts.

## Data Availability

Data is contained within the article and supplementary material

## Supplementary Materials

The following are available online at www.mdpi.com/article/10.3390/nano11102756/s1. Figure S1: PXRD and EDS analysis of NiHCF NCs; Figure S2: The surface area analysis of the NiHCF mesocrystals; Figure S3: Surface area analysis of mesoframes; Figure S4: PXRD analysis of NiHCF mesocrystal-derived mesoframes; Figure S5: EDS spectrum of hollow mesoframes; FigureS6: EDS mapping of one NiHCF mesocrystal; Figure S7: FTIR-spectra of hollow mesoframes and NiHCF mesocrystals; Figure S8: XPS analysis of NiHCF mesocrystals; Figure S9: PMS-activated degradation of the contaminant rhodamine-B under standard conditions; Figure S10: Analysis of the degradation mechanism by using scavenger molecules; Figure S11: Exploration of radical species that affect the degradation reaction of rhodamine-B; Figure S12: Analysis of the catalyst-loading, PMS amount as well as the pH and temperature impact on the degradation efficiency of rhodamine-B by using mesoframes as catalyst; Figure S13: Influence of Mesoframe catalyst loading on the degradation of rhodamine-B; Figure S14: Exploration of the PMS dosage influence on the degradation; Figure S15: Effect of pH value on the degradation of rhodamine-B; Figure S16: Rate constants obtained from the pH variation experiments; Figure S17: Temperature influence on the rhodamine-B degradation; Figure S18: PMS-activated degradation of bisphenol-A performed under standard conditions; Figure S19: Investigation of degradation mechanism by monitoring the radical species affecting the degradation; Figure S20: Investigation of the catalyst-loading, PMS dosage, pH adjustment and temperature influence on the degradation efficiency of bisphenol-A applying mesoframes as catalyst; Figure S21: Effect of catalyst-loading on the degradation of bisphenol-A; Figure S22: Influence of the PMS dosage on the removal efficiency using Mesoframes as catalyst; Figure S23: Influence of pH value on the degradation of bisphenol-A; Figure S24: Rate constants of the bisphenol-A degradations at different pH values and Figure S25: Temperature influence on the Mesoframe-catalyzed bisphenol-A degradation. Table S1: EDS data of the NiHCF mesocrystals, Table S2: EDS data of the NiHCF mesocrystal-derived Mesoframes; Table S3: Comparison of the rhodamine-B degradation conditions and the corresponding rate constants; Table S4: Comparison of the bisphenol-A degradation conditions and the corresponding rate constants; Table S5: Comparison of the bisphenol-A degradation in this work with previous reported approaches; Table S6: EDS data of the Mesoframes after the rhodamine-B catalysis and Table S7: EDS data of the Mesoframes after the bisphenol-A catalysis.
