# Supplementary material for: Environmentally Benign Formation of Nickel Hexacyanoferrate-Derived Mesoframes for Heterogeneous Catalysis
Source: Nanomaterials (Basel). 2021 Oct 18;11(10):2756. doi: 10.3390/nano11102756 (PMC8537782; doi:10.3390/nano11102756)
Supplement: Supplementary file 1 [file nanomaterials-11-02756-s001.zip › nanomaterials-1429004-supplementary.pdf]

nanomaterials

Environmentally Benign Formation of Nickel Hexacyanoferrate-Derived Mesoframes for Heterogeneous Catalysis

Sascha Keßler [1], Elrike R. Reinalter [1], Johannes Schmidt [2] and Helmut Cölfen [1,*]

Nickel Hexacyanoferrate Mesocrystal Structure and Composition

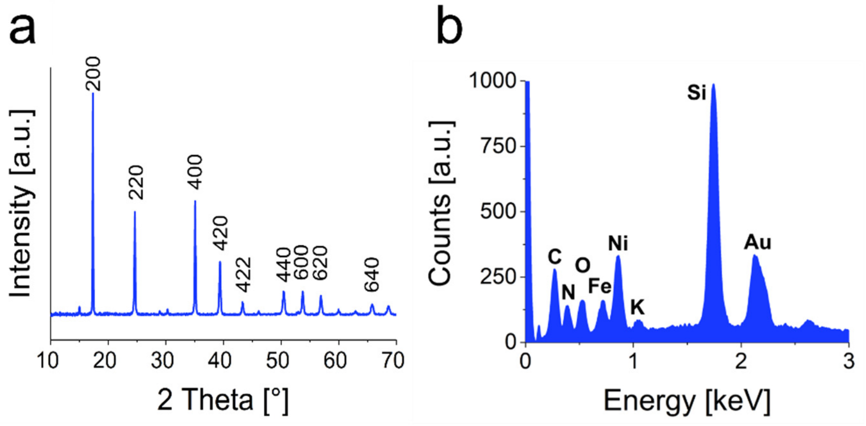

Figure S1. (**a**) Normalized PXRD pattern of NiHCF mesocrystals assigned with its respective reflexes (reference: file JCP2.2CA: 01-082-2283). (**b**) EDS spectrum of NiHCF mesocrystals with a corresponding [Ni]:[Fe] ratio of 1.24. The signals for silicon and gold occurred due to the Si(100)-wafer and the gold coating of the sample.

Table S1. EDS data of the NiHCF mesocrystals. The calculated oxygen content can be ascribed to the presence of water molecules attached to the surface.

Elements	Wt%	σWt%
**C**	18.00	0.22
**N**	12.67	0.17
**O**	7.97	0.06
**K**	2.35	0.06
**Fe**	15.83	0.28
**Ni**	20.65	0.22

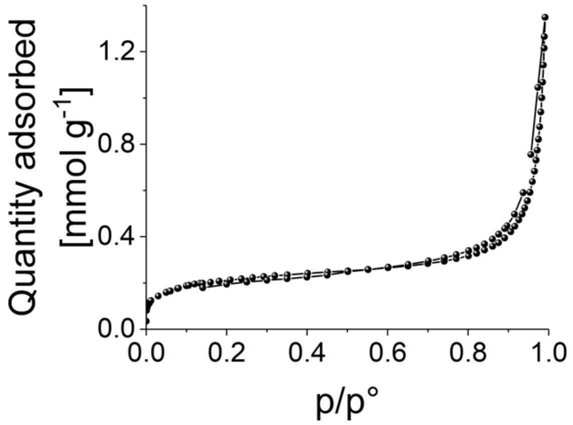

Figure S2. The surface area analysis of the NiHCF mesocrystals by nitrogen isotherm measurements. The data were processed by means of the BET theory. The surface area was calculated to be 35 $m^2\ g^{-1}$.

Mesoframe Characterization

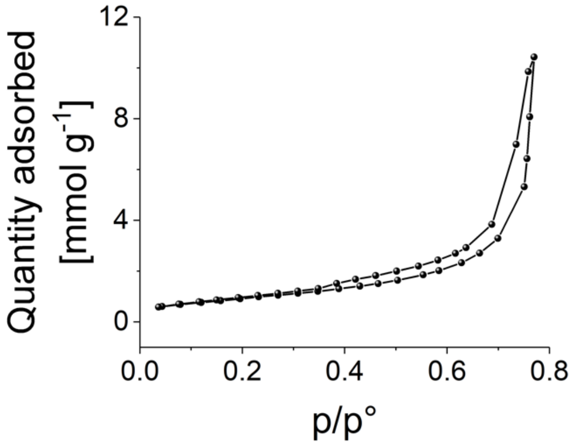

Figure S3. Surface area analysis of Mesoframes obtained after alkaline etching. Characterization was conducted by using nitrogen isotherm measurements. The data were processed by means of the BET theory. The surface area was calculated to be 96 $m^2\ g^{-1}$.

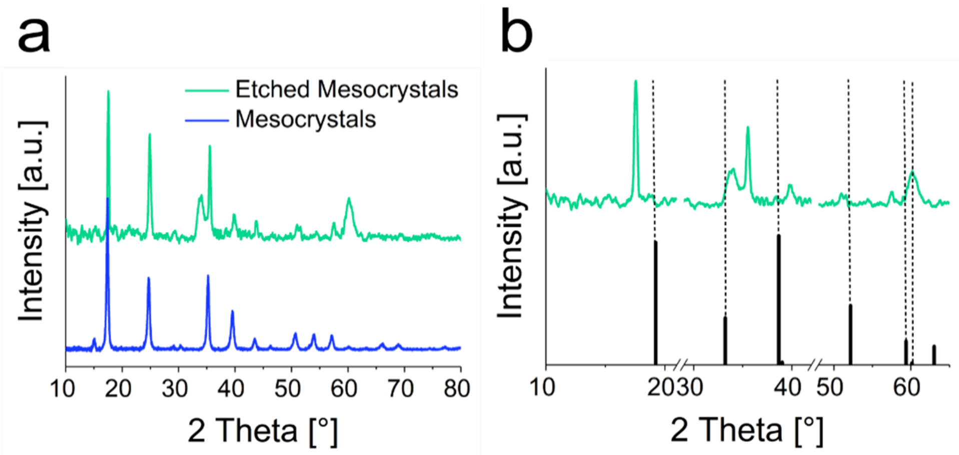

Figure S4. PXRD analysis of NiHCF mesocrystal-derived mesoframes. Normalized PXRD patterns of (**a**) mesoframes and NiHCF mesocrystals. (**b**) Normalized PXRD pattern of the hollow mesoframes focusing on the special β-$Ni(OH)_2$ reference reflexes.

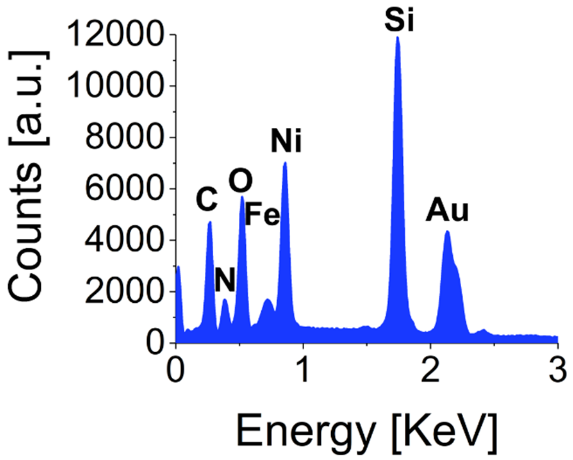

Figure S5. EDS spectrum of hollow mesoframes. The [Ni]:[Fe] ratio was calculated to be 4.06. The respective gold and silicon signals can be attributed to the gold coating and the Si (100)-wafer onto which the sample was drop-cast.

Table S2. EDS data of the NiHCF mesocrystal-derived Mesoframes.

Elements	Wt%	σWt%
C	11.57	0.32
N	4.70	0.24
O	9.64	0.093
K	1.30	0.093
Fe	5.07	0.41
Ni	21.61	0.32

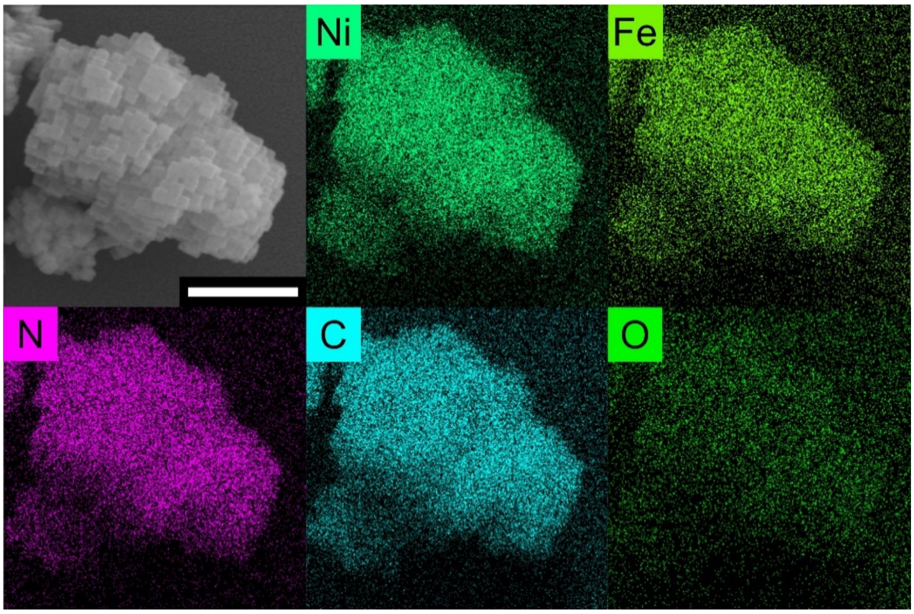

Figure S6. EDS mapping of one NiHCF mesocrystal showing the elements (Ni, Fe, N, C and O) within the superstructure.

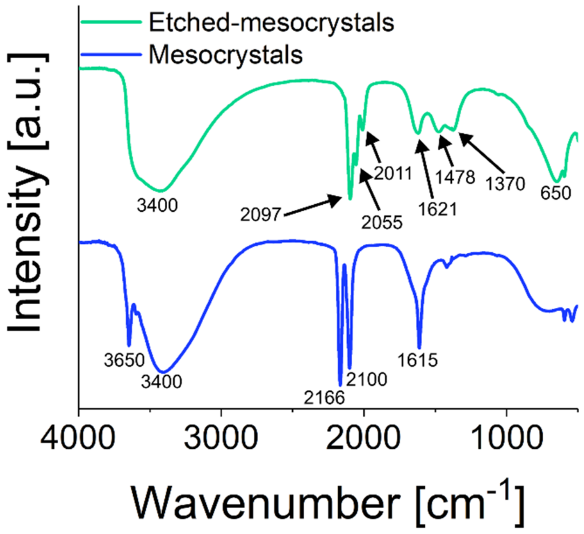

Figure S7. FTIR-spectra of hollow mesoframes and NiHCF mesocrystals (measured in transmission mode).

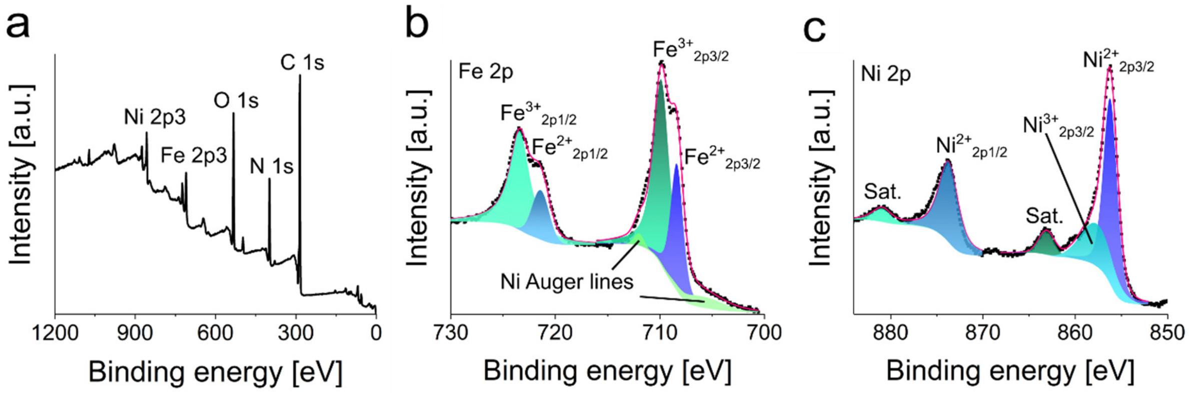

Figure S8. XPS analysis of NiHCF mesocrystals. (**a**) XPS total survey of the mesocrystals. (**b**) Fe 2p and (**c**) Ni 2p spectra of the NiHCF mesocrystals.

Investigation of the PMS-Activated Rhodamine-B Degradation

In the case of catalyst-loading, the increase from 0.1 g L^{-1} to 1.0 g L^{-1} led to a gradual increase in the degradation efficiency (Figure S12a). The rate constant was also increased from 0.41 min^{-1} to 0.58 min^{-1} (Figure S13, table S3). For the PMS dosage, the use of only 0.1 g L^{-1} led to a reduced efficiency while an increased PMS of 0.7 g L^{-1} and 1.5 g L^{-1} initiated the decomposition of the mesoframes and thus an even lower degradation reaction occurred (Figure S12b, figure S14 and table S3). The decomposition of the catalyst was indicated by a rapid color change in the beginning of the reaction from green to black. These results showed that the selected amount of PMS was already in the optimal range.

In terms of the pH impact on the degradation efficiency, higher pH values seem to be more beneficial for the efficient removal of rhodamine-B. At a pH of 3, only reduced catalytic activity was observed. This reduced activation of PMS under acidic conditions is characteristic due to the higher stability of the PMS at low pH values. By adjusting the pH from 5, 7 and 9 to 11, the rate constant could be increased up to a value of 0.73 min^{-1} (Figure S12c, figure S15 and S16, table S3). For practical use, the application of such a high pH value makes less sense. Wastewater usually has a pH value in the range between 5 and 9. Nevertheless, an increased efficiency for the removal of rhodamine-B was also obtained in this range, which makes an application in wastewater treatment attractive.

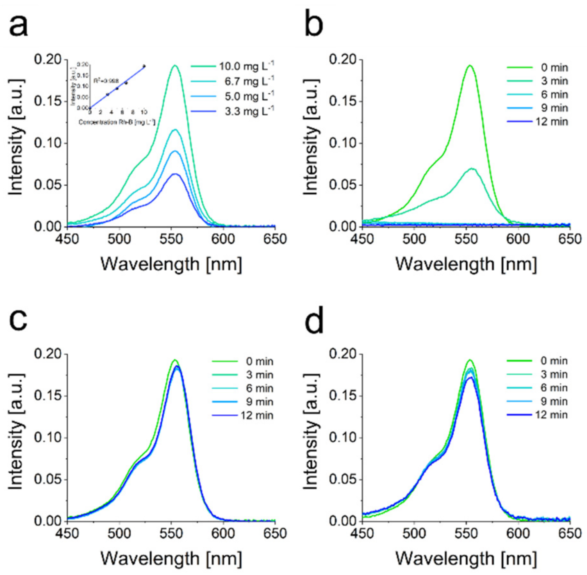

Figure S9. PMS-activated degradation of the contaminant rhodamine-B under standard conditions. (**a**) Calibration of the UV-Vis spectra and determination of the extinction coefficient. UV-Vis absorption spectra of rhodamine-B at different concentrations. UV-Vis spectra of rhodamine-B degradation in the presence of (**b**) Co_3O_4, (**c**) of NiHCF mesocrystals and (**d**) only PMS. Reaction conditions: [Rhodamine-b] = 10.0 mg L^{-1}, V = 100 mL, T = 25 °C, pH = 5, [Mesoframe] = 0.10 g L^{-1}, [PMS] = 0.30 g L^{-1}.

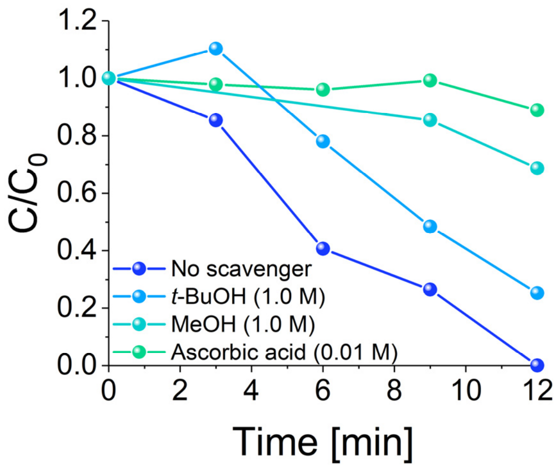

Figure S10. Analysis of the degradation mechanism by using scavenger molecules such as *t*-BuOH, MeOH and AA. Reaction conditions: [Rhodamine-b] = 10.0 mg L^{-1}, V = 100 mL, T = 25 °C, pH = 5, [Mesoframe] = 0.10 g L^{-1}, [PMS] = 0.30 g L^{-1}.

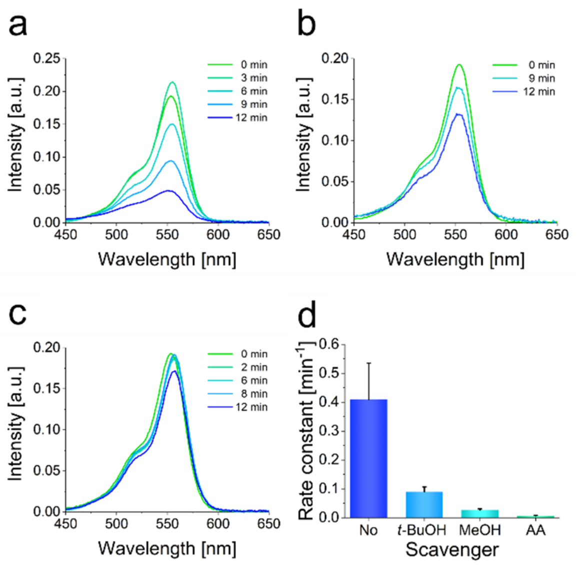

Figure S11. Exploration of radical species that affect the degradation reaction of rhodamine-B. *t*-BuOH, MeOH and AA were used as scavenger molecules. UV-Vis absorption spectra of rhodamine-B removal using (**a**) *t*-BuOH (1.00 mol L^{-1}) as scavenger for hydroxy radicals, (**b**) MeOH (1.00 mol L^{-1}) for sulfate radicals and (**c**) AA (0.01 mol L^{-1}) for hydroxy and sulfate radicals. (**d**) Rate constants of the corresponding removal reactions. Reaction conditions: [Rhodamine-b] = 10.0 mg L^{-1}, V = 100 mL, T = 25 °C, pH = 5, [Mesoframe] = 0.10 g L^{-1}, [PMS] = 0.30 g L^{-1}.

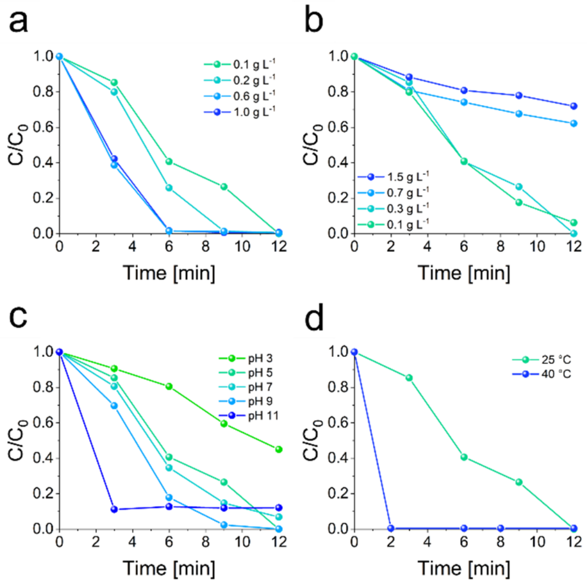

Figure S12. Analysis of the catalyst-loading, PMS amount as well as the pH and temperature impact on the degradation efficiency of rhodamine-B by using mesoframes as catalyst. (**a**) Impact of catalyst loading on the degradation. Reaction conditions: [Rhodamine-b] = 10.0 mg L^{-1}, V = 100 mL, T = 25 °C, pH = 5, [Mesoframe] = varied, [PMS] = 0.3 g L^{-1}. (**b**) PMS dosage affecting the degradation efficiency. Reaction conditions: [Rhodamine-b] = 10.0 mg L^{-1}, V = 100 mL, T = 25 °C, pH = 5, [Mesoframe] = 0.10 g L^{-1}, [PMS] = varied. (**c**) Effect of pH value on degradation reaction. Reaction conditions: [Rhodamine-B] = 10.0 mg L^{-1}, V = 100 mL, T = 25 °C, pH = varied, [Mesoframe] = 0.10 g L^{-1}, [PMS] = 0.30 g L^{-1}. (**d**) Temperature influence on degradation efficiency. Reaction conditions: [Rhodamine-B] = 10.0 mg L^{-1}, V = 100 mL, T = 25 °C or 40 °C, pH = 5, [Mesoframe] = 0.10 g L^{-1}, [PMS] = 0.30 g L^{-1}.

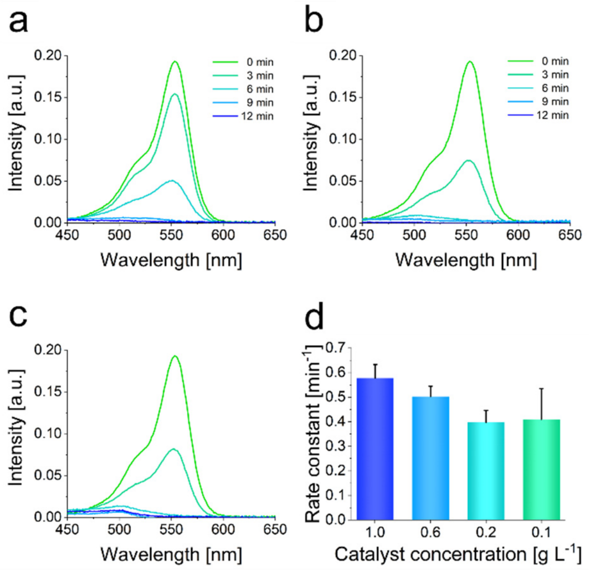

Figure S13. Influence of mesoframe catalyst loading on the degradation of rhodamine-B. UV-Vis absorption spectra of the rhodamine-B degradation in the presence of (**a**) 0.20 g L^{-1}, (**b**) 0.60 g L^{-1} and (**c**) 1.00 g L^{-1} mesoframes. (**d**) Rate constants of the corresponding degradation. Reaction conditions: [Rhodamine-b] = 10.0 mg L^{-1}, V = 100 mL, T = 25 °C, pH = 5, [Mesoframe] = varied, [PMS] = 0.3 g L^{-1}.

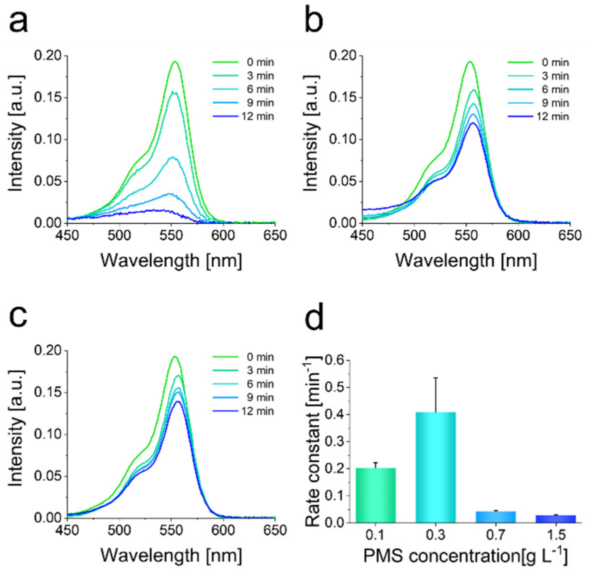

Figure S14. Exploration of the PMS dosage influence on the degradation. UV-Vis absorption spectra of rhodamine-B degradation in the presence of (**a**) 0.10 g L^{-1}, (**b**) 0.70 g L^{-1} and (**c**) 1.50 g L^{-1}. (**d**) Rate constants obtained from the UV-Vis spectra. Reaction conditions: [Rhodamine-b] = 10.0 mg L^{-1}, V = 100 mL, T = 25 °C, pH = 5, [Mesoframe] = 0.10 g L^{-1}, [PMS] = varied.

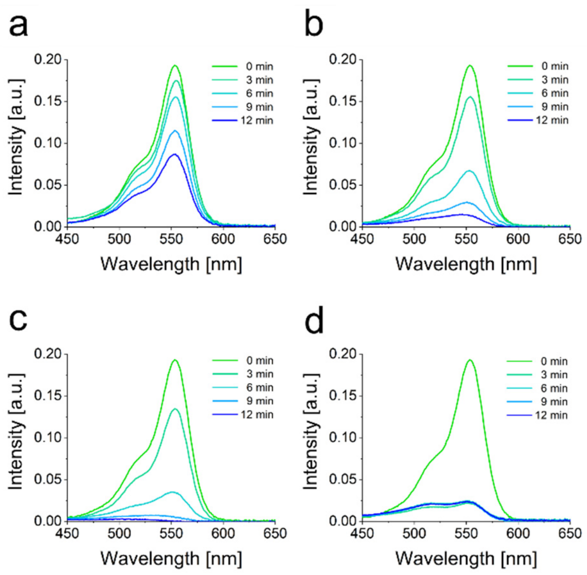

Figure S15. Effect of pH value on the degradation of rhodamine-B. UV-Vis absorption spectra of the rhodamine-B degradation in the presence of a pH value of (**a**) 3, (**b**) 7, (**c**) 9 and (**d**) 11. Reaction conditions: [Rhodamine-B] = 10.0 mg L^{-1}, V = 100 mL, T = 25 °C, pH = varied, [Mesoframe] = 0.10 g L^{-1}, [PMS] = 0.30 g L^{-1}.

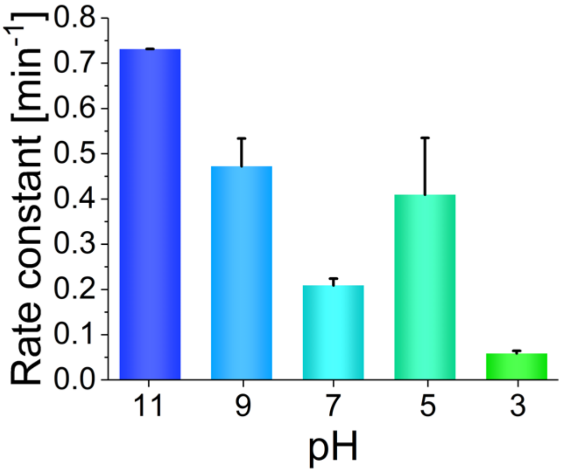

Figure S16. Rate constants obtained from the pH variation experiments. Reaction conditions: [Rhodamine-B] = 10.0 mg L^{-1}, V = 100 mL, T = 25 °C, pH = varied, [Mesoframe] = 0.10 g L^{-1}, [PMS] = 0.30 g L^{-1}.

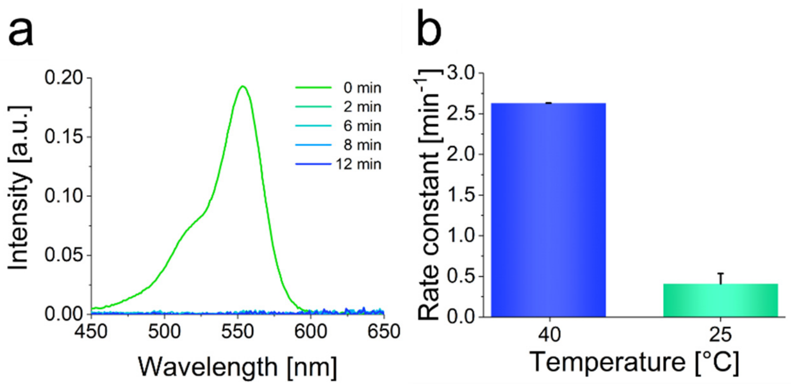

Figure S17. Temperature influence on the rhodamine-B degradation. (**a**) UV-Vis absorption spectrum of the rhodamine-B removal at 40 °C. (**b**) Corresponding rate constants of the temperature-dependent degradation. Reaction conditions: [Rhodamine-B] = 10.0 mg L^{-1}, V = 100 mL, T = 25 °C or 40 °C, pH = 5, [Mesoframe] = 0.10 g L^{-1}, [PMS] = 0.30 g L^{-1}.

Table S3. Comparison of the rhodamine-B degradation conditions and the corresponding rate constants.

Catalyst	Conditions					Data		
	$C_{rhodamine\text{-}B}$ [mg L⁻¹]	$C_{Catalyst}$ [g L⁻¹]	C_{PMS} [g L⁻¹]	pH	T [°C]	Time [min]	Removal [%]	Rate constant k [min⁻¹]
**None**	10	0.00	0.30	5	25	12	11	0.01
**Co_3O_4**	10	0.10	0.30	5	25	12	100	0.61
**Mesocrystals**	10	0.10	0.30	5	25	12	4	0.01
**Mesoframes**	10	0.10	0.30	5	25	12	100	0.41
**Mesoframes**	10	0.10	0.30	5	25	6	59	0.41
**Mesoframes**	10	0.20	0.30	5	25	6	74	0.40
**Mesoframes**	10	0.60	0.30	5	25	6	98	0.50
**Mesoframes**	10	1.00	0.30	5	25	6	98	0.58
**Mesoframes**	10	0.10	0.10	5	25	12	94	0.20
**Mesoframes**	10	0.10	0.30	5	25	12	100	0.41
**Mesoframes**	10	0.10	0.70	5	25	12	38	0.04
**Mesoframes**	10	0.10	1.50	5	25	12	28	0.03
**Mesoframes**	10	0.10	0.30	3	25	12	55	0.06
**Mesoframes**	10	0.10	0.30	5	25	12	100	0.41
**Mesoframes**	10	0.10	0.30	7	25	12	93	0.21
**Mesoframes**	10	0.10	0.30	9	25	12	100	0.47
**Mesoframes**	10	0.10	0.30	11	25	12	88	0.73
**Mesoframes**	10	0.10	0.30	5	25	3	15	0.41
**Mesoframes**	10	0.10	0.30	5	40	2	100	2.63
**Mesoframes[1]**	10	0.10	0.30	5	25	12	75	0.09
**Mesoframes[2]**	10	0.10	0.30	5	25	12	31	0.03
**Mesoframes[3]**	10	0.10	0.30	5	25	12	11	0.01

[1] **Degradation in the presence of *t*-BuOH (1.00 mol L⁻¹).**
[2] **Degradation in the presence of MeOH (1.00 mol L⁻¹).**
[3] **Degradation in the presence of AA (0.01 mol L⁻¹).**

Investigation of the PMS-activated bisphenol- degradation

The variation of the catalyst concentration between 0.2 g L⁻¹ and 2.0 g L⁻¹ revealed a similar gradual increase in efficiency as for the rhodamine-B removal (Figure S20a). The efficiency was clearly affected, as rate constants up to 2.25 min⁻¹ could be derived (Figure S21, Table S4). Certainly, the presence of an increased amount of catalytically active material and the associated exposure of active sites led to the enhancement of the degradation efficiency.

The investigation of the PMS dosage showed similar results to the degradation of rhodamine-B, leading to the conclusion that an excess of PMS leads to the decomposition of the catalyst. The results clearly show that the amount of PMS must be chosen sensitively. In the interval from 0.2 g L⁻¹ and 0.4 g L⁻¹, the degradation efficiency increased somewhat, but between 1.0 g L⁻¹ and 2.0 g L⁻¹ it decreased drastically (Figure S20b, Figure S22 and Table S4).

The impact of the pH value on the degradation reaction appears to be one of the key features for the practical application, as it relies upon the varying pH value of wastewater. The degradation of bisphenol-A follows the same pattern as for rhodamine-B. The efficiency increases significantly with increasing pH value. The rate constants increase from 0.24 min⁻¹ at a pH of 3 to a 4.4 times higher rate constant of 1.06 min⁻¹ at a pH of 11 (Figure S20c, S23 and S24; Table S4). Although the highest efficiency is achieved at a pH of 11, the efficiency in the moderate pH range between 5 and 9 is also relatively high, which accommodates the pH fluctuations of wastewater.

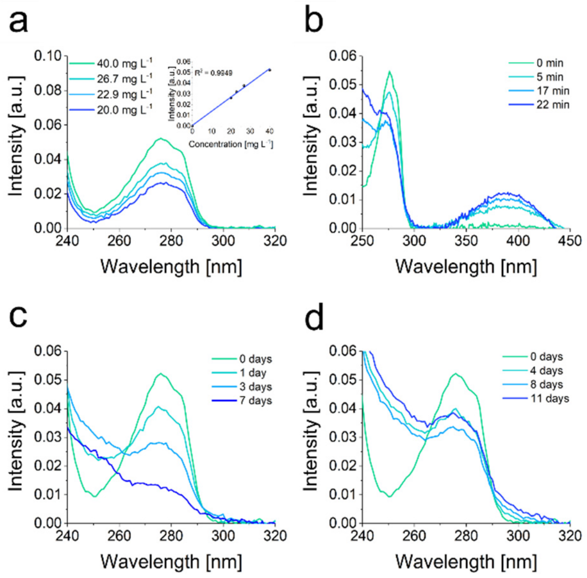

Figure S18. PMS-activated degradation of bisphenol-A performed under standard conditions. (**a**) Determination of the extinction coefficient of bisphenol-A. UV-Vis absorption spectra of bisphenol-A at different calibration concentrations. UV-Vis spectra of the bisphenol-A degradation in the presence of (**b**) Co_3O_4, (**c**) of NiHCF mesocrystals and (**d**) only PMS. Reaction conditions: [Bisphenol-A] = 40.0 mg L^{-1}, V = 100 mL, T = 25 °C, pH = 7, [Mesoframe] = 0.20 g L^{-1}, [PMS] = 0.20 g $L^{-1.}$

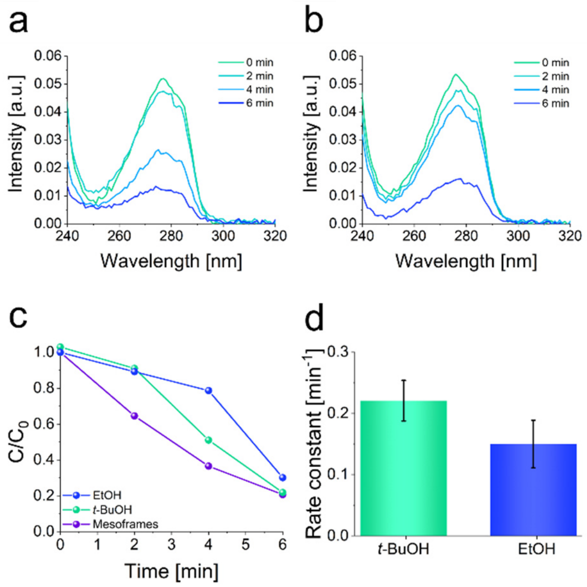

Figure S19. Investigation of degradation mechanism by monitoring the radical species affecting the degradation. *t*-BuOH and EtOH were used as scavengers. UV-Vis absorption spectrum of the bisphenol-A degradation in the presence of (**a**) *t*-BuOH (1.00 mol L^{-1}) as scavenger for hydroxy radicals, (**b**) EtOH (1.00 mol L^{-1}) for sulfate and hydroxy radicals. (**c**) Degradation profiles obtained from the UV-Vis spectra. (**d**) Respective rate constants. Reaction conditions: [Bisphenol-A] = 40.0 mg L^{-1}, V = 100 mL, T = 25 °C, pH = 7, [Mesoframe] = 0.20 g L^{-1}, [PMS] = 0.20 g $L^{-1.}$

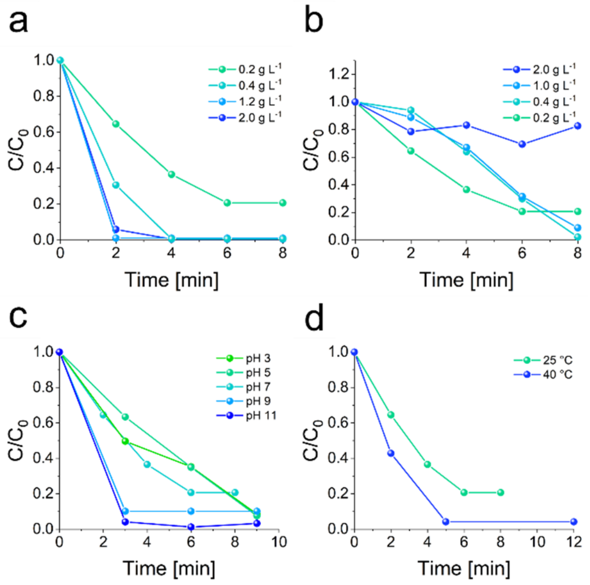

Figure S20. Investigation of the catalyst-loading, PMS dosage, pH adjustment and temperature influence on the degradation efficiency of bisphenol-A, applying mesoframes as catalyst. (**a**) Effect of catalyst loading on the degradation reaction. Reaction conditions: [Bisphenol-A] = 40.0 mg L^{-1}, V = 100 mL, T = 25 °C, pH = 7, [Mesoframe] = varied, [PMS] = 0.20 g L^{-1}. (**b**) Impact of the PMS dosage on the degradation efficiency. Reaction conditions: [Bisphenol-A] = 40.0 mg L^{-1}, V = 100 mL, T = 25 °C, pH = 7, [Mesoframe] = 0.20 g L^{-1}, [PMS] = varied. (**c**) Influence of pH value on degradation reaction. Reaction conditions: [Bisphenol-A] = 40.0 mg L^{-1}, V = 100 mL, T = 25 °C, pH = varied, [Mesoframe] = 0.20 g L^{-1}, [PMS] = 0.20 g L^{-1}. (**d**) Temperature impact on the degradation efficiency. Reaction conditions: [Bisphenol-A] = 40.0 mg L^{-1}, V = 100 mL, T = 25 °C or 40 °C, pH = 7, [Mesoframe] = 0.20 g L^{-1}, [PMS] = 0.20 g L^{-1}.

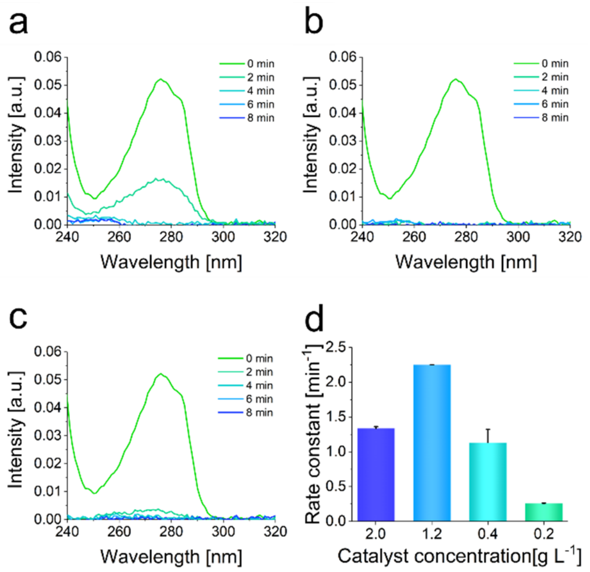

Figure S21. Effect of catalyst-loading on the degradation of bisphenol-A. UV-Vis absorption spectra of the bisphenol-A removal in the presence of (**a**) 0.40 g L^{-1}, (**b**) 1.20 g L^{-1} and (**c**) 2.00 g L^{-1} Mesoframes. (**d**) Rate constants obtained from the corresponding UV-Vis spectra. Reaction conditions: [Bisphenol-A] = 40.0 mg L^{-1}, V = 100 mL, T = 25 °C, pH = 7, [Mesoframe] = varied, [PMS] = 0.20 g L^{-1}.

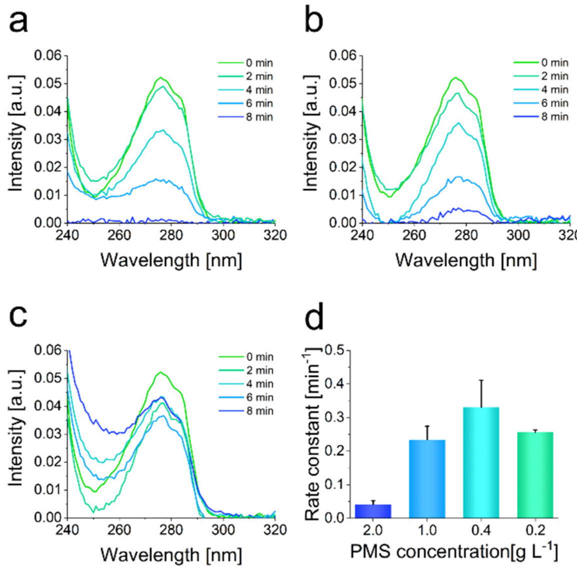

Figure S22. Influence of the PMS dosage on the removal efficiency using Mesoframes as catalyst. UV-Vis absorption spectra of bisphenol-A degradation reaction in the presence of (**a**) 0.40 g L^{-1}, (**b**) 1. 00 g L^{-1} and (**c**) 2.00 g L^{-1}. (**d**) Corresponding rate constants. Reaction conditions: [Bisphenol-A] = 40.0 mg L^{-1}, V = 100 mL, T = 25 °C, pH = 7, [Mesoframe] = 0.20 g L^{-1}, [PMS] = varied.

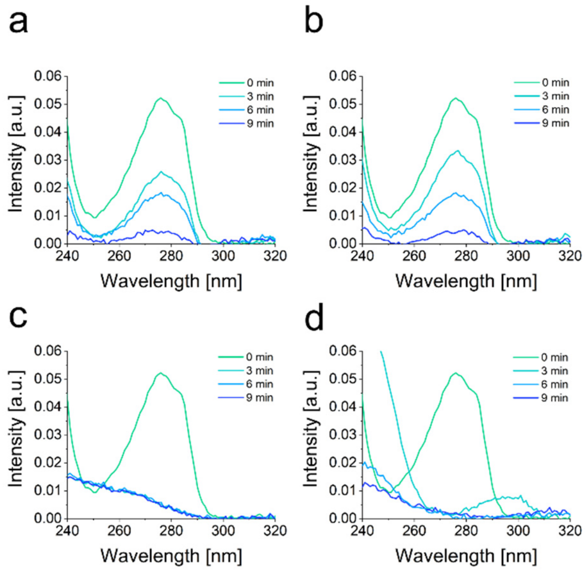

Figure S23. Influence of pH value on the degradation of bisphenol-A. UV-Vis absorption spectra of the bisphenol-A degradation in the presence of a pH value of (**a**) 3, (**b**) 5, (**c**) 9 and (**d**) 11. Reaction conditions: [Bisphenol-A] = 40.0 mg L^{-1}, V = 100 mL, T = 25 °C, pH = varied, [Mesoframe] = 0.20 g L^{-1}, [PMS] = 0.20 g L^{-1}.

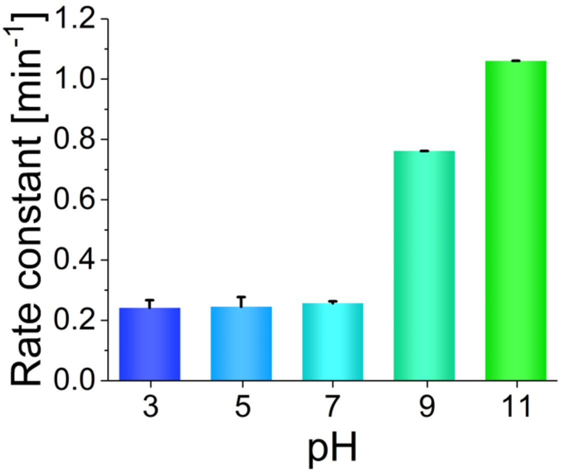

Figure S24. Rate constants of the bisphenol-A degradations at different pH values. Reaction conditions: [Bisphenol-A] = 40.0 mg L^{-1}, V = 100 mL, T = 25 °C, pH = varied, [Mesoframe] = 0.20 g L^{-1}, [PMS] = 0.20 g L^{-1}.

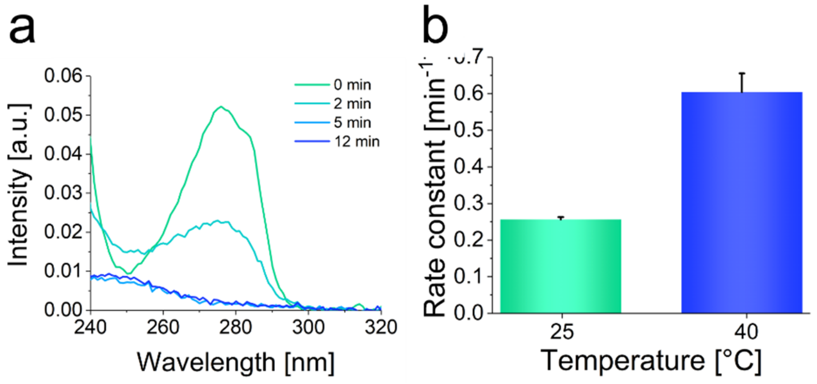

Figure S25. Temperature influence on the Mesoframe-catalyzed bishphenol-A degradation. (**a**) UV-Vis absorption spectrum of the bisphenol-A degradation at 40 °C. (**b**) Respective rate constants of the temperature-dependent degradation. Reaction conditions: [Bisphenol-A] = 40.0 mg L^{-1}, V = 100 mL, T = 25 °C or 40 °C, pH = 7, [Mesoframe] = 0.20 g L^{-1}, [PMS] = 0.20 g L^{-1}.

Table S4. Comparison of the bisphenol-A degradation conditions and the corresponding rate constants.

Catalyst	Conditions						Data	
	$C_{Bisphenol\text{-}A}$ [mg L^{-1}]	$C_{Catalyst}$ [g L^{-1}]	C_{PMS} [g L^{-1}]	pH	T [°C]	Time [min]	Removal [%]	Rate constant k [min^{-1}]
**None**	40	0.00	0.20	7	25	8	0	0.00
**Co_3O_4**	40	0.20	0.20	7	25	8	14	0.02
**Mesocrystals**	40	0.20	0.20	7	25	8	0	0.00
**Mesoframes**	40	0.20	0.20	7	25	8	80	0.26
**Mesoframes**	40	0.20	0.20	7	25	2	35	0.26
**Mesoframes**	40	0.40	0.20	7	25	2	70	1.13
**Mesoframes**	40	1.20	0.20	7	25	2	99	2.25
**Mesoframes**	40	2.00	0.20	7	25	2	94	1.34
**Mesoframes**	40	0.20	0.20	7	25	8	81	0.26
**Mesoframes**	40	0.20	0.40	7	25	8	98	0.33
**Mesoframes**	40	0.20	1.00	7	25	8	91	0.23
**Mesoframes**	40	0.20	2.00	7	25	8	27	0.04
**Mesoframes**	40	0.20	0.20	3	25	3	50	0.24
**Mesoframes**	40	0.20	0.20	5	25	3	37	0.25
**Mesoframes**	40	0.20	0.20	7	25	4	63	0.26
**Mesoframes**	40	0.20	0.20	9	25	3	100	0.76
**Mesoframes**	40	0.20	0.20	11	25	3	97	1.06

**Mesoframes**	40	0.20	0.20	7	25	6	80	0.41
**Mesoframes**	40	0.20	0.20	7	40	5	96	0.61
**Mesoframes[1]**	40	0.20	0.20	7	25	6	78	0.22
**Mesoframes[2]**	40	0.20	0.20	7	25	6	70	0.15

[1] Degradation in the presence of *t*-BuOH (1.00 mol L^{-1}).
[2] Degradation in the presence of EtOH (1.00 mol L^{-1}).

Table S5. Comparison of the bisphenol-A degradation in this work with previous reported approaches.

**Catalyst**	**Conditions**				**Data**			
	**Catalyst synthesis[1] [°C]**	**$C_{Bisphenol-A}$ [mg L^{-1}]**	**$C_{Catalyst}$ [g L^{-1}]**	**C_{PMS} [g L^{-1}]**	**Time [min]**	**Removal [%]**	**Rate constant [min^{-1}]**	**Ref.**
**MnFeO@C**	300	20	0.2	No data	30	95	0.10	1
**$Fe_xMn_{6-x}Co_4$–N@C**	650	20	0.1	0.2	10	100	0.48	2
**$Mn_{1.8}Fe_{1.2}O_4$**	400	10	0.2	0.2	15	100	0.10	3
**Fe/Co PBA**	80	20	0.2	0.2	4	100	1.52	4
**Fe/Co PBA/PAN**	80	20	0.233	0.5	240	94	No data	5
**$Fe_xCo_{3-x}O_4$**	500	20	0.1	0.2	60	95	0.05	6
**NiFe Layered Double Hydroxide**	150	100	0.5	0.4	15	~ 90	0.29	7
**Mesoframes**	25	40	0.20	0.20	8	80	0.26	This work

[1] Temperature during the synthesis of the respective catalyst material.

Table S6. EDS data of the Mesoframes after the rhodamine-B catalysis.

**Elements**	**Wt%**	**σWt%**
**C**	16.01	0.22
**N**	7.52	0.17
**O**	20.4	0.06
**K**	0.85	0.06
**Fe**	6.00	0.28
**Ni**	27.82	0.22

Table S7. EDS data of the Mesoframes after the bisphenol-A catalysis.

**Elements**	**Wt%**	**σWt%**
**C**	36.75	0.19
**N**	6.30	0.14
**O**	18.87	0.06
**Na**	0.08	0.03
**K**	0.71	0.06
**Fe**	3.098	0.24
**Ni**	12.89	0.19
**Cu**	12.75	0.32

References

1. Yang, Y.; Li, Y.; Hong, P.; Wu, Z.; Xie, C.; Zhang, K.; Lianxiang, L.; He, J.; Kong, L.; Liu, J. Surface-active MnFeO@C cubes as enhanced peroxymonosulfate activators for efficient degradation of bisphenol A. *Appl. Surf. Sci.*, **2021**, *538*, 14808.
2. Li, X.; Ao, Z.; Liu, J.; Sun, H.; Rykov, A. I.; Wang, J. Topotactic Transformation of Metal-Organic Frameworks to Graphene-Encapsultated Transition-Metal Nitrides as Efficient Fenton-like Catalysts. *ACS Nano* **2016**, *10*, 11532–11540.
3. Huang, G-X.; Wang, C-Y.; Yang, C-W.; Guo, P-C.; Yu, H-Q. Degradation of Bisphenol A by Peroxymonosulfate Catalytically Activated with $Mn_{1.8}Fe_{1.2}O_4$ Nanospheres: Synergism between Mn and Fe. *Environ. Sci. Technol.* **2017**, *51*, 12611–12618.
4. Zhang, W.; Zhang, H.; Yan, X.; Zhang, M.; Luo, R.; Qi, J.; Sun, X.; Shen, J.; Han, W.; Wang, L.; et al. Controlled synthesis of bimetallic Prussian blue analogues to activate peroxymonosulfate for efficient bisphenol A degradation. *J. Hazard. Mater.* **2019**, *387*, 121701.

5. Wang, H.; Wang, C.; Qi, J.; Yan, Y.; Zhang, M.; Yan, X.; Sun, X.; Wang, L.; Li, J. Spiderweb-Like Fe-Co Prussian Blue Analogue Nanofibers as Efficient Catalyst for Bisphenol-A Degradation by Activating Peroxymonosulfate. *Nanomaterials* **2019**, *9*, 402.
6. Li, X.; Wang, X.; Zhang, B.; Rykov, A.I.; Ahmed, M.A.; Wang, J. $Fe_xCo_{3-x}O_4$ nanocages derived from nanoscale metal-organic frameworks for removal of bisphenol A by activation of peroxymonosulfate. *Appl. Catal. B* **2016**, *181*, 788–799.
7. Yue, D.; Yan, X.; Guo, C.; Qian, X.; Zhao, Y. NiFe Layered Double Hydroxide (LDH) Nanosheet Catalyst with Fe as Electron Transfer Mediator for Enhanced Persulfate Activation. *J. Phys. Chem. Lett.* **2020**, *11*, 968–973.
